# Vesiculobullous Cutaneous Larva Migrans in the Absence of Domestic Dogs and Cats. Successful Treatment with Oral Ivermectin

**DOI:** 10.3390/tropicalmed9050106

**Published:** 2024-05-07

**Authors:** Manuel Calvopina, Karla Lozano-Alvarez, Sandra Enriquez-Morillo, Ignacio Cordova-Calisto

**Affiliations:** 1 One Health Research Group, Facultad de Medicina, Universidad de las Américas, P.O. Box 17-17-9788, Quito 170513, Ecuador; karla.lozano@udla.edu.ec (K.L.-A.); ignacio.cordova@udla.edu.ec (I.C.-C.); 2 Instituto de Investigación en Zoonosis (CIZ), Universidad Central del Ecuador, P.O. Box 17-03-100, Quito 170129, Ecuador; ienriquez@uce.edu.ec

**Keywords:** cutaneous larva migrans, wild animals, hookworms, Ecuador

## Abstract

While conducting research in a protected ecological reserve within Ecuador’s subtropical rainforest, a 49-year-old biologist, residing in an Andean city, contracted hookworm-related cutaneous larva migrans (Hr-CLM) in the vesiculobullous clinical form. Since there were no domestic dogs or cats in the reserve, it is likely that wild animals carrying *Ancylostoma* sp. larvae infected the patient. She was effectively treated with two doses of oral ivermectin, administered 31 days after getting the infection. This case was diagnosed in a temperate city; therefore, a comprehensive travel history and clinical assessments are crucial for an accurate diagnosis and timely treatment.

## 1. Introduction

Cutaneous larva migrans (CLM) or “creeping eruption” is a zoonosis caused by animal hookworms. Hookworm-related CLM (Hr-CLM) distinguishes itself from other cutaneous helminthiases such as *Strongyloides stercoralis* (“larva currens”) and others [1]. The WHO recognizes it as a Neglected Tropical Disease (NTD) under ‘Scabies and other ectoparasitosis’ [2]. Hr-CLM results from skin penetration of hookworm larvae present in the soil. These larvae originate from eggs expelled in the feces of infected domestic dog or cat with hookworms, primarily *Ancylostoma braziliense* or *A. caninum*. Other animal hookworms such as *Uncinaria stenocephala* and *Bunostomum phlebotomum* can also be agents of Hr-CLM [1,3]. Moreover, wild animals, including sylvatic canids, felids, ursids, procyonids, and mustelids, can be infected with *A. caninum*, while sylvatic felids and mustelids can be infected by *A. braziliense*, potentially transmitting the infection to humans [4,5]. In recent times, *A. ceylanicum*, a hookworm found in both sylvatic and domestic canids, has been reported to infect humans in the Americas, including Ecuador [6].

These zoonotic hookworms responsible for CLM are predominantly found in tropical and subtropical regions across Southeast Asia, Africa, South America, and the Caribbean, and they have been recently reported in the United States, Canada, and Europe [7,8]. Hr-CLM is the most frequent travel-associated skin disease of tropical origin [3,9], and it is likely to be a common disease in local residents, even if underreported. In less developed regions, free-roaming dogs and cats often have high rates of hookworm infection (https://www.cdc.gov/parasites/zoonotichookworm/epi.html (accessed on 12 February 2024).

Ecuador is in the northwestern part of South America; bordered by the Pacific Ocean, it encompasses three ecoregions. In the tropical Coastal and Amazon regions, Hr-CLM is commonly diagnosed [10]. However, in the temperate Andes region, occurrences are rare and often poorly diagnosed and treated. A recent study revealed that 19.4% of free-roaming dogs in Pacific beaches harbor hookworms [11]. Ecuador, being a megadiverse country, boasts abundant wildlife, especially in subtropical and tropical forests where wild felids, canines, olingos, and procyonids have been recorded [12,13].

Diagnosis of Hr-CLM relies on clinical presentation in individuals with recent travel to tropical regions, beach exposure, or engagement in soil-related activities. Within two to six days following this, intensely pruritic, elevated, serpiginous reddish-brown tracks appear as the larvae migrate at a rate of 1 or 2 centimeters per day, reaching lengths of up to 15 to 20 cm. Some patients may develop vesiculobullous lesions and folliculitis [1]. Hr-CLM mostly occurs on the lower extremities. The distinctive clinical features of the “creeping trail” (length, width, migration speed, location, duration) aid in the differential diagnosis. No further tests are currently recommended, and there are no specific serological or culture methods available [3]. Eosinophilia is found in less than 40% of patients with CLM, but it is also not specific [1].

The lesions resolve spontaneously within 2 to 8 weeks, although longer durations have been documented, and they may recur days to months later or persist in follicles [14]. Anthelminthic therapy helps relieve symptoms and reduce bacterial superinfection. Ivermectin (200 mcg/kg orally once daily for one or two days) is the preferred drug. Albendazole (400 mg orally for three days) represents an alternative treatment [15]. Ivermectin taken in a single dose is well tolerated and highly efficacious, with cure rates of 94% to 100%. Albendazole is also effective and well tolerated at a regimen of 400 to 800 mg/d (according to weight) for 3 days [3]. Both drugs are listed in the WHO essential medicines. Topical agents are an alternative treatment but can be challenging to obtain in endemic areas. Antihistamines assist in managing pruritus, while topical corticosteroids are beneficial for patients with severe allergic reactions.

The aim of reporting this case is to raise awareness among health personnel regarding its diagnosis, particularly among travelers returning from regions without the presence of domestic dogs and cats.

## 2. Case Report

A 49-year-old female biologist, resident of Quito and engaged in university teaching and research, presented with a serpiginous dermal lesion located on the back of her right forearm. It began approximately 31 days before consultation; it initially manifested as a small reddish papule and gradually grew, irregular in shape and accompanied by intense itching, which caused sleep disturbances. This lesion appeared 5 days after her return to Quito (incubation period) from a 2-day visit to a protected ecological reserve situated in the coastal subtropical jungle (Longitude: −78.877605, Latitude: 0.165901). She participated in field activities involving cleaning the ground before setting Shannon traps on tree trunks to collect sandflies. The patient suspected cutaneous leishmaniasis (CL) and sought a laboratory for scraping and smear tests; however, they were not performed as the lesion was not ulcerated. Five years ago, she experienced CL on her left upper eyelid, which was successfully treated with meglumine antimoniate injections.

**Figure 1:**
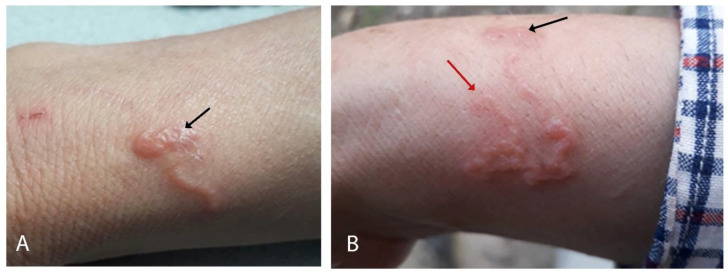
(**A**) Photograph of the skin lesion observed 7 days after onset, highlighting the site of penetration of the *Ancylostoma* sp. larva (black arrow). (**B**) The vesiculobullous lesion, upon receiving ivermectin, displays a snake-like progression, and the final active and expanding site is indicated (red arrow).

On physical examination, no fever or apparent pathology in any other system or organ was noted. A skin lesion was observed on the dorsum of her right forearm, displaying a serpiginous path approximately 19 cm in length. The initial point appeared less inflamed compared to the endpoint. The tract exhibited erythema, elevation, and vesicles, yet no signs of ulceration were present, consistent with a diagnosis of vesiculobullous lesions characteristic of Hr-CLM. There were no signs of bacterial infection, dermographism, burrows, or lymphadenopathy (Figure 1A,B). No other tests were performed. Additionally, her cat underwent a coproparasitic test, yielding negative results for intestinal parasites.

**Figure 2:**
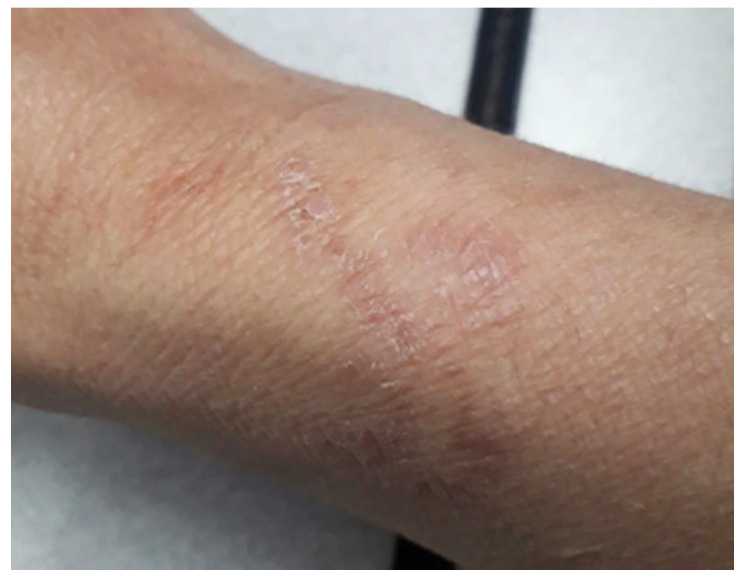
The healing serpiginous tract five days after ivermectin.

The patient was treated with ivermectin (200 mcg/kg/day) for two consecutive days, and no adverse effects related to the medication were observed. Within 24 h after treatment, the itching and the lesion growth stopped. After 5 days of ivermectin, the inflammation subsided, and peeling occurred without leaving a scar (Figure 2).

Written informed consent was obtained from the patient for the publication of her case.

## 3. Discussion

Typically, the larvae of intestinal hookworms infecting domestic dogs and cats, such as *Ancylostoma braziliense*, *A. caninum*, and *Uncinaria stenocephala*, are the causative agents for Hr-CLM [1,3]. However, in this case, Hr-CLM was diagnosed in a person conducting activities within a protected subtropical natural reserve in Ecuador, where domestic dogs and cats are absent. This observation leads us to suspect that wild canids and felids inhabiting the ecoregion might be harboring hookworm species responsible for causing CLM in humans. Sylvatic canids, felids, ursids, procyonids, and mustelids can also be infected with *A. caninum*, while sylvatic felids and mustelids can be infected with *A. braziliense* [4,5]. Numerous wild animals, such as Bush dogs (*Speothos venaticus*), Coatis (*Nasu narica*), Olingos (*Bassaricyon gabbii*), Kinkajou (*Potos flavus*), Greater grison (*Galictis vittata*), Otters (*Lontra longicaudis*), various species of “Tigrillos” (*Leopardus* spp.), Jaguars (*Panthera onca*), and other Tayra species, are recorded in the mentioned Ecuadorian ecoregion [12,13]. Additionally, other animal hookworms, such as *Uncinaria stenocephala* and *Bunostomum phlebotomum*, known to infect wild animals, might also act as potential agents for Hr-CLM [3].

Furthermore, it is unclear whether *A. tubaeforme,* found in felids, or *A. ceylanicum,* found in wild canids and felids, can infect humans and induce CLM. In the perspective of One Health, future studies are advised to characterize hookworm species in wildlife hosts and their environment. Despite the substantial impact of these hookworms on domestic animals, wildlife, and human health, studies addressing wildlife infectious diseases and their implications remain limited. This paucity of research underscores the neglected status of these diseases, highlighting the need for increased attention and investigations. 

While Hr-CLM is categorized as a tropical disease and frequently reported among returning travelers [3], the diagnosis in this case was established in Quito, an Andean city. Therefore, a comprehensive travel history and clinical assessments are crucial for an accurate diagnosis and timely treatment. The present case, an academic professional, contracted the infection during research activities, which is evidence to classify it as an occupational disease. Workers exposed to soil contaminated by feces of domestic and wild animals (which are potential definitive hosts of hookworms), such as breeders, farmers, agriculturists, and gardeners, should be considered at a high risk of infection. Consequently, Hr-CLM may warrant consideration as a potential occupational disease [7].

It is prudent to advise physicians to remain vigilant about Hr-CLM and other tropical diseases, even in temperate regions because of global warming [16]. Reports indicate cases of Hr-CLM caused by *A. braziliense* and *U. stenocephala* from European and North American countries [7,8]. The case presented here acquired the infection in a subtropical region—elevation 550–1400 m—while most reported cases have been linked to travelers visiting beaches [9].

Typically, Hr-CLM primarily localizes in the feet and legs due to barefoot walking, accounting for more than 90% of cases. In our case, it was observed on the forearm 5 days after returning to Quito, which was directly associated with her activities in the field. The incubation period can vary based on the species involved; lesions caused by *A. braziliense* may manifest within an hour, whereas lesions caused by *U. stenocephala* may take several days to appear [1]. 

The case described herein exhibited a rapid response to systemic ivermectin. As recommended by The Medical Letter (2013) and other authors, the preferred treatment is oral ivermectin (200 mcg/kg) for one or two days. Usually, Hr-CLM is self-limiting; however, larval migration may persist for several months with potential recurrence in follicles [14]. In such a context, anthelminthic therapy is advised to alleviate symptoms and reduce the risk of bacterial superinfection [7]. While topical thiabendazole has shown efficacy, it is not available in Ecuador. Alternative topical agents, such as ivermectin, albendazole, and permethrin, although not tested in Ecuador, could be considered. Although antihistamines, corticosteroids, and topical antibiotics are advised for relieving itching, inflammation, and bacterial superinfection in certain scenarios [1], we found no need for their use in our case. 

## Data Availability

The data presented in this Case report are available on request from the corresponding author.
